# *Salvia miltiorrhiza* Bunge Regulates the Differentiation of mESCs into Cardiomyocytes via the Wnt/β-Catenin Signaling Pathway

**DOI:** 10.3390/cells15090786

**Published:** 2026-04-26

**Authors:** Guotao Lu, Qi Sun, Wei Ren, Jihong Yang, Fan Yang

**Affiliations:** 1 College of Veterinary Medicine, Northwest A&F University, Yangling 712100, China; luguotao@nwafu.edu.cn (G.L.); sunqi0914@nwafu.edu.cn (Q.S.); davidr@nwafu.edu.cn (W.R.); 2 Shaanxi Centre of Stem Cells Engineering & Technology, Northwest A & F University, Yangling 712100, China; 3 State Key Laboratory for Development and Utilization of Forest Food Resources, Zhejiang University, Hangzhou 311300, China; 4 Zhejiang Shouxiangu Pharmaceutical Co., Ltd., Wuyi 321200, China; 5 Shenzhen Research Institute, Northwest A&F University, Shenzhen 518057, China

**Keywords:** *Salvia miltiorrhiza* Bunge, Wnt/β-catenin, cardiac differentiation, tanshinone IIA, embryonic stem cell

## Abstract

**Highlights:**

**What are the main findings?**

- *Salvia miltiorrhiza* Bunge regulates mESC differentiation into cardiomyocytes in a stage-dependent manner.
- The pro-cardiogenic activity of *Salvia miltiorrhiza* Bunge is mainly mediated by suppression of canonical Wnt/β-catenin signaling, with tanshinone IIA identified as a key active constituent.

**What are the implications of the main findings?**

- *Salvia miltiorrhiza* Bunge and tanshinone IIA as natural small-molecule regulators to enhance stem cell-derived cardiomyocyte generation.
- Precise temporal modulation of Wnt/β-catenin signaling is critical for optimizing pluripotent stem cell cardiac differentiation strategies.

**Abstract:**

*Salvia miltiorrhiza* Bunge has been used traditionally for cardiovascular disorders, but its specific roles in stem cell cardiac differentiation remain unclear. In this study, we examined whether *Salvia miltiorrhiza* Bunge (SM) promotes cardiomyocyte differentiation from mouse embryonic stem cells (mESCs) and defined its underlying mechanism. To dynamically monitor cardiac differentiation, we established a *Tnnt2*-H2B-mCherry reporter mESC line that retained normal pluripotency and differentiation capacity. Using an embryoid body-based differentiation system, we found that SM exerted a distinct temporal effect on lineage progression: treatment during the early differentiation window inhibited pluripotency maintenance, proliferation, and mesodermal development, whereas administration during the cardiac precursor stage markedly enhanced cardiomyocyte formation, as indicated by increased beating embryoid bodies and upregulation of *Isl1*, *Nkx2.5*, *Tnnt2*, *Myh6*, and *Myl7*. Mechanistically, transcriptomic and protein analyses showed that SM suppressed canonical Wnt/β-catenin signaling, including downregulation of *Dvl2*, *β-catenin*, *Axin2*, *c-Myc*, and *Cyclin D1*, while Wnt activation WAY262611 partially reversed these effects. Further compound screening identified tanshinone IIA (Tan IIA) as the principal active constituent of SM, which largely recapitulated the pro-cardiogenic and Wnt-inhibitory effects of the crude extract. Together, these findings identify SM and Tan IIA as stage-dependent regulators of mESC fate and support their potential utility in natural product-based strategies for improving stem cell-derived cardiomyocyte generation.

## 1. Introduction

Cardiovascular diseases (CVDs) are the foremost causes of death and disability globally, primarily due to the limited regenerative capacity of adult cardiomyocytes following injury, such as myocardial infarction [1,2,3]. During a myocardial infarction, extensive necrosis and apoptosis of myocardial cells occur, resulting in their replacement by fibrous scar tissue, thus causing irreversible heart function damage [4,5,6]. This irreversible loss of functional cardiac tissue culminates in heart failure, highlighting an urgent need for strategies to regenerate myocardium.

Embryonic stem cells (ESCs) including pluripotent and totipotent stem cells, possess the abilities of unlimited self-renewal and multi-directional differentiation, making them an ideal source for myocardial regeneration [7,8,9]. Under appropriate culture conditions in vitro, ESCs directed induction can yield large numbers of highly purified cardiomyocytes, which serve for cell transplantation, disease modeling, and drug screening [10,11,12]. However, directing PSC differentiation efficiently and specifically toward functional cardiomyocytes requires precise temporal modulation of key developmental signaling pathways. Among these pathways, the Wnt/β-catenin signaling plays a biphasic and stage-specific role during cardiogenesis [13,14,15,16]. Its activation is crucial for initial exit from pluripotency and induction of the mesodermal lineage. Subsequently, timely inhibition of Wnt/β-catenin signaling is essential for the specification of cardiac progenitor cells (CPCs) and their maturation into cardiomyocytes [13,14,17,18]. Small-molecule inhibitors of this pathway, such as IWP compounds, are routinely used in established differentiation protocols to enhance cardiac yield [19,20]. This delicate regulatory dynamic underscore the importance of identifying compounds.

Traditional Chinese medicines have been explored as a rich source of bioactive compounds with pleiotropic effects. *Salvia miltiorrhiza* Bunge, a prominent traditional Chinese medicine known for enhancing blood circulation and alleviating blood stasis, is extensively used in treating cardiovascular conditions like coronary heart disease and angina pectoris [21]. Modern pharmacological studies reveal that SM and its active components, such as tanshinone I (Tan I), tanshinone IIA (Tan IIA) and salvianolic acid B (Sal B), offer various cardioprotective benefits, including antioxidative, anti-inflammatory, and anti-apoptotic effects, along with promoting angiogenesis and improving microcirculation [22]. Research indicates that SM or its active ingredients might directly influence stem cell regulation [23,24], particularly in promoting the differentiation of human placenta-derived mesenchymal stem cells into cardiomyocytes. Despite SM’s long-standing use in cardiovascular medicine and its well-documented cardioprotective effects, its role and mechanisms in directing the myocardial differentiation of stem cells remain insufficiently understood.

In this study, we explored how SM and its active compound(s) influence the differentiation of mESCs into cardiomyocytes. SM demonstrates stage-specific function during ESC-derived cardiomyocyte induction. SM was found to inhibit mesoderm development during the early differentiation phase. However, in later stages, it promotes the expression of transcription factors like *Isl1* and *Nkx2.5* in cardiac progenitor cells, thereby influencing cardiomyocyte differentiation. Our findings also indicate that SM and Tan IIA regulate these processes by inhibiting the Wnt/β-catenin signaling pathway.

## 2. Materials and Methods

### 2.1. Chemicals and Reagents

Tan IIA (IT0530), Tan I (IT0520), Sal B (IS1940), Sal A (SS8090), LA (SL8220) and Cry (IC0590) were obtained from Solarbio (Beijing, China). All active compound purity ≥ 98%. The alkaline phosphatase staining kits (BCBT0082 and MKCD0656) were procured from Sigma-Aldrich (St. Louis, MO, USA); MTT(purity above 98%, ST1537), the ethynyl-labeled deoxyuridine (EdU) Cell Proliferation Kit with Alexa Fluor 555 (C0075) were acquired from Beyotime (Shanghai, China); Additionally fetal bovine serum (FBS,10091148), Dulbecco’s Modified Eagle’s Medium (11965092), NEAA (11140050), GlutaMAX supplement (35050061), β-mercaptoethanol (21985023), penicillin-streptomycin (15140122) were sourced from Gibco (Carlsbad, CA, USA). WAY262611(purity above 99%, S9828) were sourced from Selleck (Houston, TX, USA).

Antibodies against Pou5f1 (ET1612-20, 1:1000), Sox2 (HA721155, 1:1000), Nanog (ET1610-2, 1:1000), alpha actinin (ER1803-60, 1:1000), GAPDH (ET1702-66, 1:1000) and Brachyury (ET7109-35, 1:1000) were sourced from HUABIO (Hangzhou, China). Antibodies for Pcna (AWA10303, 1:1000), Cyclin D1 (AWA10518, 1:2000), Islet 1 (AWA10163, 1:500) and Nkx2.5 (AWA64024, 1:1000) were procured from Abiowell (Changsha, China). GSK-3β (F1042, 1:1000), β-catenin (F2521, 1:5000), Wnt3a (F1056, 1:1000) and Dvl2 (F0572, 1:1000) antibodies were purchased from Selleck (Houston, TX, USA). SSEA-1 (SC-101462, 1:1000) and *Tnnt2* (SC-20025, 1:2000) antibodies were acquired from Santa Cruz Biotechnology (Dallas, TX, USA). c-Myc (T55150F, 1:1000) and β-Tubulin (M30109F, 1:5000), β-Actin (P30002, 1:5000) antibodies were obtained from Abmart (Shanghai, China), Axin2 (20540-1-AP, 1:1000) antibodies was obtained from Proteintech Group, Inc ( Rosemont, IL, USA). Alexa-594 and Alexa-488 conjugated anti-rabbit and anti-mouse secondary antibodies (1:500) were purchased from ZSGB-Bio (Beijing, China), and HRP-conjugated secondary antibodies (1:8000) were from BOSTER Biological Technology (Wuhan, China).

### 2.2. Cell Culture

The mESCs (R1) were maintained in high-glucose DMEM supplemented with 15% FBS, 1 μmol/L non-essential amino acids, 1 mmol/L GlutaMAX supplement, 100 μmol/L β-mercaptoethanol, 100 U/mL penicillin, 100 mg/mL streptomycin, and 1000 U/mL leukemia inhibitory factor (LIF). Cultures were passaged every two days, and only cells in the exponential growth phase were used for subsequent experiments.

### 2.3. Cell Differentiation

Differentiation of mESCs followed the published protocol [25]. Briefly, embryoid bodies (EBs) were formed and harvested after 2 days, then transferred into ultralow-attachment plates for 3 days of suspension culture. EBs were thereafter plated on gelatin-coated dishes for adherent culture, with medium changes every 2 days. Differentiation was induced using differentiation medium supplemented with 0.5 mg/mL SM. Differentiation outcomes and cellular function were assessed by RT-qPCR and RNA sequencing, immunofluorescence, and Western blotting.

### 2.4. Dimethylthiahiazo (-z-y1)-3,5-Di-phenytetrazoliumromide (MTT) Assay

The MTT assay was carried out as previously described [26]. mESCs were plated in 96-well plates at a density of 5 × 10^3^ cells per well. The SM was diluted to the desired concentrations using culture medium before being applied to the cells. Following treatment, the cells were incubated at 37 °C for 24 h. After incubation, the medium was removed, and 10 μL of MTT solution (5 mg/mL) was added to each well, followed by a 4 h incubation. The MTT solution was then aspirated, and 100 μL of DMSO was added to dissolve the formazan crystals. Absorbance at 570 nm was measured using a SpectraMax M5 microplate reader (Molecular Devices, Sunnyvale, CA, USA).

### 2.5. EdU Incorporation Assay

The EdU incorporation assay was performed as previously described [27]. Briefly, mESCs were treated with SM at indicated concentrations for 48 h. Incorporation of EdU into newly synthesized DNA was assessed using the manufacturer’s kit and protocol. All samples were examined by fluorescence microscopy. Proliferation was quantified as the proportion of EdU-positive cells determined by flow cytometry. Nuclei were counterstained with DAPI for 5 min at room temperature.

### 2.6. Transient Transfection

*Tnnt2* targeting vectors were kindly provided by Dr. Cai Chenleng (Guangzhou Institutes of Biomedicine and Health, Chinese Academy of Sciences). mESCs at 80–90% confluence were transiently transfected with 1.25 µg of *Tnnt2* targeting vectors and 1.25 µg of PX459-*Tnnt2* sgRNA expression vector using Lipofectamine 2000, following the manufacturer’s protocol (Invitrogen, Carlsbad, CA, USA). Six hours after transfection, the medium was replaced with complete medium. Forty-eight hours after transfection, positively transfected cells were selected with 500 µg/mL G418 and 1 µg/mL puromycin. PCR was performed with PrimerSTAR DNA Polymerase. Two long primer pairs (F1 + R1, F2 + R2) were used to screen for positive clones, and two short primer pairs (F3 + R3, F4 + R4) were used to further validate positive clone #8. PCR products were confirmed by DNA sequencing. The sgRNA and PCR primers are listed in Table 1.

### 2.7. Western Blot Analysis

As previously described [28], cells were homogenized on ice in RIPA lysis buffer containing 1% protease and phosphatase inhibitors. Proteins were extracted by centrifugation at 12,000× *g* for 15 min, and concentrations were determined by BCA assay. Samples were separated on 10% SDS–PAGE and transferred to polyvinylidene difluoride (PVDF) membranes. Membranes were blocked with 5% skim milk and then incubated overnight at 4 °C with the indicated primary antibody and horseradish peroxidase–conjugated secondary antibodies were used for detection.

### 2.8. RT-qPCR Analysis

RNA extraction and RT-qPCR were performed as described previously [29]. Total RNA was isolated from cells using TRIzol reagent (Thermo Fisher, Waltham, MA, USA) and treated with DNase I to remove potential genomic DNA contamination. cDNA was synthesized with a commercial cDNA Synthesis Kit following the manufacturer’s instructions (Vazyme, Nanjing, China). RT-qPCR was then carried out using FastStart SYBR Green Master (Vazyme, Nanjing, China). Relative expression levels were calculated with the 2^−△△CT^ method, using β-actin transcripts for internal normalization. RT-qPCR primer sequences are listed in Table 2.

### 2.9. Immunocytochemical Staining Analysis

Alkaline phosphatase (AP) activity was measured using an AP staining kit (Sigma-Aldrich, St. Louis, MO, USA) according to the manufacturer’s instructions. Immunostaining was performed as previously described [30]. Briefly, cells were fixed with 4% paraformaldehyde and permeabilized with 0.3% Triton X-100 (Sigma, St. Louis, MO, USA). After blocking in 10% normal goat serum (Vector Laboratories, Burlingame, CA, USA), samples were incubated overnight at 4 °C with primary antibodies against Oct4 (1:500, HUABIO, Hangzhou, China), Sox2 (1:200, HUABIO, Hangzhou, China), SSEA1 (1:200, Santa Cruz Biotechnology, Dallas, TX, USA), *Tnnt2* (1:200, Santa Cruz Biotechnology, Dallas, TX, USA), and Brachyury (1:100, HUABIO, Hangzhou, China). Primary antibodies were detected with Alexa Fluor 594- or 488-conjugated secondary antibodies. Nuclei were counterstained with DAPI. Images were acquired on a ZEISS microscope (Carl Zeiss AG, Oberkochen, Germany).

### 2.10. RNA-Seq Analysis

For the analysis of RNA-seq data, clean reads were aligned to the mm10 mouse genome utilizing HISAT2. Expression matrices and RPKM values were computed with Feature Counts. Differential gene expression among samples was assessed using DESeq2, while heatmaps were produced with the R package pheatmap (R version 4.3.1).

### 2.11. Statistical Analysis

Statistical analyses were conducted with GraphPad Prism version 10.0 (GraphPad Software, San Diego, CA, USA). Quantitative results are reported as mean ± S.D. Comparisons used an unpaired two-tailed Student’s *t*-test or one-way ANOVA with Tukey’s correction for multiple comparisons. Differences were considered statistically significant at *p* < 0.05.

## 3. Results

### 3.1. Establishment of Tnnt2-H2B mCherry Reporter Line and Differentiation into Cardiomyocytes

**Figure 1:**
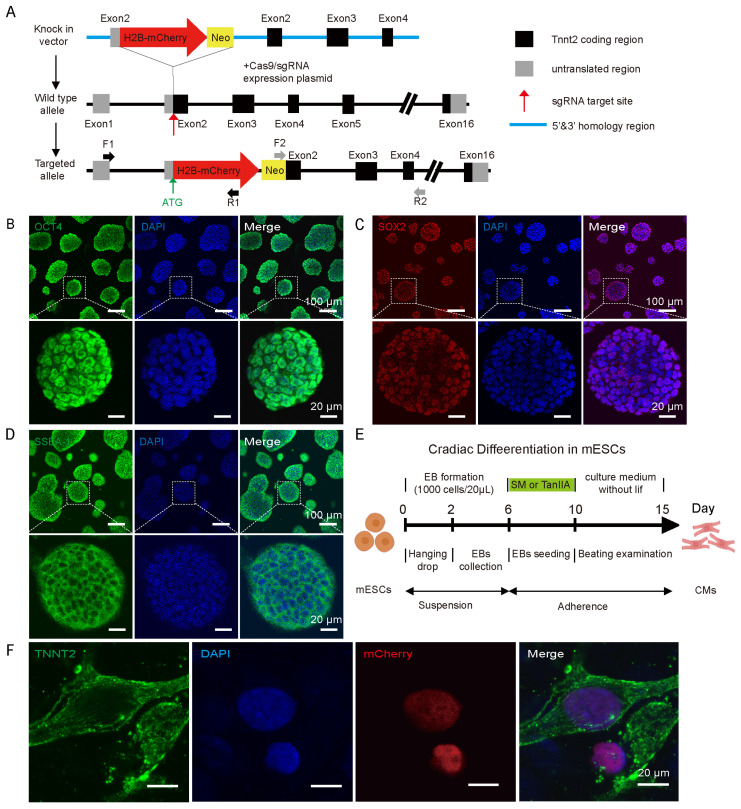
Establishment of *Tnnt2*-H2B-mCherry reporter line and its differentiation into cardiomyocytes. (**A**) Schematic of the knock-in strategy. Primer sets F1/R1 and F2/R2 were used for genotyping. (**B**) Immunofluorescence confirming expression of OCT4 in the reporter line. (**C**) Immunofluorescence staining results for SOX2 in the reporter cell lines. (**D**) Immunofluorescence staining for SSEA-1 in the reporter cell line Insets show magnified views. Scale bars: 100 μm (main), 20 μm (insets). (**E**) Schematic of the directed cardiomyocyte differentiation protocol. (**F**) TNNT2 staining of differentiated cardiomyocytes on day 15. Scale bar: 20 μm.

Cardiomyocyte differentiation is a tightly controlled developmental process [31,32,33]. To explore SM’s impact on the differentiation timeline and functionality of cardiomyocytes, we generated a reporter mESC line by knocking in an H2B-mCherry cassette at the *Tnnt2* locus to visualize and track cardiomyocytes (Figure 1A). *Tnnt2* (also known as *cTnT*) encodes cardiac troponin T, a gene that is silenced in pluripotent cells and activated during cardiac differentiation [34]. Comprehensive genomic validation confirmed the correct integration of the reporter gene construct at both the 5′ and 3′ ends (Figure S1A–C). Importantly, the gene modification did not compromise pluripotency, as evidenced by the unchanged cell morphology, alkaline phosphatase activity and expression levels of *Pou5f1 (Oct4)*, *Sox2*, *Ssea-1*, and *Nanog* in the reporter gene cell line (Figure 1B–D and Figure S1D,E). To validate the reporter line’s developmental capacity, we applied a classical hanging drop embryoid body differentiation protocol to generate cardiomyocytes (Figure 1E). Time-course expression analysis showed dynamic, stage-specific changes in lineage markers. Pluripotency genes (*Oct4*, *Sox2*) declined over time, while mesoderm (*Brachyury/T*), cardiac mesoderm (*Mesp1*, *Eomes*), and cardiac precursor markers (*Gata4*, *Isl1*) were transiently upregulated. *Tnnt2* expression increased progressively (Figure S1F). These results demonstrate that cells transit through mesoderm and cardiac precursor states before acquiring a cardiomyocyte identity. Accordingly, we partitioned the differentiation timeline into four consecutive stages: mESC (days 0–2), mesoderm (days 3–5), cardiac precursor (days 6–10), and cardiomyocyte (days 11–15). Nuclear localization of mCherry, DAPI fluorescence and *Tnnt2* immunostaining provide direct visual confirmation of cardiomyocyte transitions (Figure 1F). Together, these findings indicate that the reporter system effectively monitors the expression of the cardiomyocyte specialization marker *Tnnt2*.

### 3.2. SM Inhibits the Pluripotency Maintenance and Cell Proliferation in mESCs

**Figure 2:**
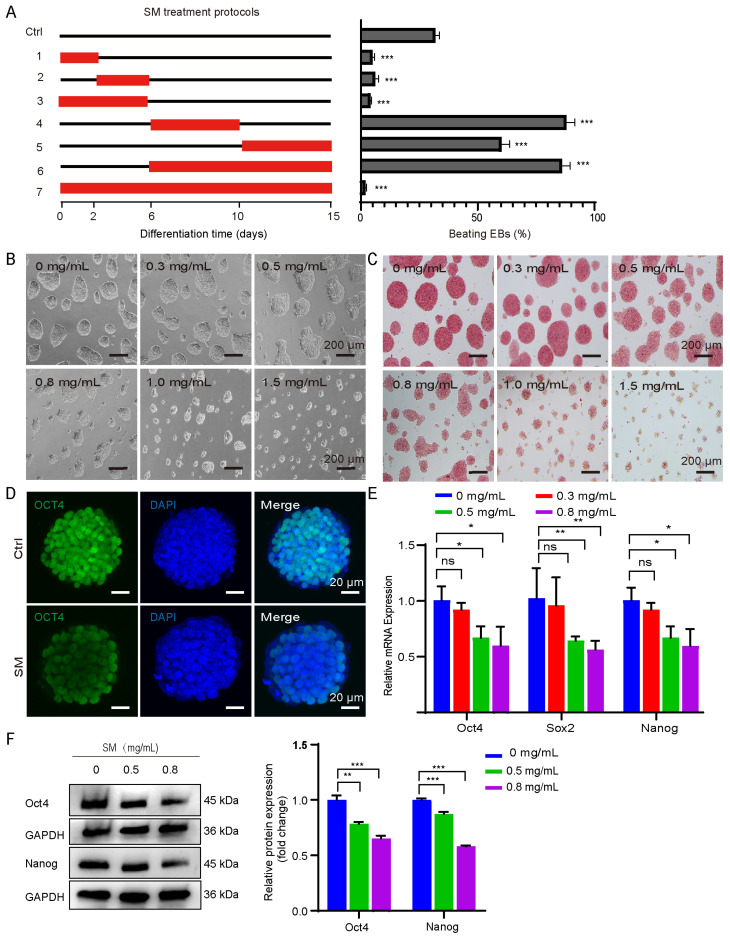
SM inhibits pluripotency maintenance of mESCs. (**A**) Experimental timeline for SM treatment and its effect on differentiation efficiency. Data are mean ± S.D. (*n* = 3). *** *p* < 0.001 vs. control (Ctrl). (**B**) Morphology of mESCs treated with SM. Scale bar: 200 μm. (**C**) Alkaline phosphatase staining of mESCs treated with SM. Scale bar: 200 μm. (**D**) Immunofluorescence of Oct4 after SM treatment (0.5 mg/mL, 48 h). Scale bar: 20 μm. (**E**) Relative mRNA expression of pluripotency genes. (**F**) Protein expression levels of Oct4 and Nanog detected by Western blot analysis and corresponding quantification. Data are mean ± S.D. ns, not significant, * *p* < 0.05, ** *p* < 0.01, *** *p* < 0.001.

We next assessed how SM affects cardiac differentiation efficiency by introducing SM (0.5 mg/mL) at different time points according to well established differentiation protocol (Figure 2A). SM treatment notably enhanced cardiomyocyte differentiation between days 6 and 10, as shown in Figure 2A, similar to the effects observed during the D6–15 differentiation period. By day 15, nearly 90% of EBs exhibited rhythmic contractions. Interestingly, adding SM during days 0–2, 3–5, or 0–5 significantly reduced the proportion of beating EBs. The results imply that SM may exert a dual role in regulating cardiomyocyte differentiation. To assess whether SM alters expression of pluripotency genes in mESCs, we exposed undifferentiated mESCs to increasing SM concentrations and observed marked changes in cell morphology and number (Figure 2B,C). Simultaneously, alkaline phosphatase activity declined significantly after SM treatment compared with the control (Figure 2C). The core pluripotency transcription factors *Oct4*, *Sox2*, and *Nanog* decreased gradually as SM concentration increased. Immunofluorescence confirmed this loss of pluripotency (Figure 2D), and quantitative mRNA and protein analyses confirmed the downregulation (Figure 2E–F). Together, these results show that SM disrupts mESC pluripotency by suppressing key markers at both the transcriptional and translational levels.

During the study, we found that the number of cells gradually decreased as the concentration of SM increased (Figure 2B,C). Therefore, we examined its impact on cell proliferation. Cell counting experiments revelated that SM treatment significantly and dose-dependently reduced the cell proliferation rate (Figure S2A), consistent with MTT assay results. (Figure S2B). Both mRNA and protein levels of proliferative cell nuclear antigen (Pcna), essential for the S-phase as an extension factor of DNA polymerase, were significantly downregulated (Figure S2C–E). At the transcriptional level, *Cyclin A*, which regulates the G1/S transition and drives the S-phase, also showed a significant decrease (Figure S2C). To identify specific cell cycle defects causing growth arrest, we performed an EdU incorporation experiment. Immunofluorescence and flow cytometry analyses demonstrated a significant reduction in the EdU-positive cell population following SM treatment (Figure S2F,G). Quantitative analysis showed a decrease in the percentage of EdU-positive cells from 75% in the control group to 40% in the 0.5 mg/mL SM treatment group (Figure S2G), suggesting significant impairment in DNA replication. In subsequent experiments, due to proliferation inhibition issues, we did not use SM at concentrations of 0.8–1.5 mg/mL. Based on the above findings, SM inhibits the pluripotency maintenance and proliferation in mESCs.

### 3.3. SM Inhibits the Induction of the Mesoderm

**Figure 3:**
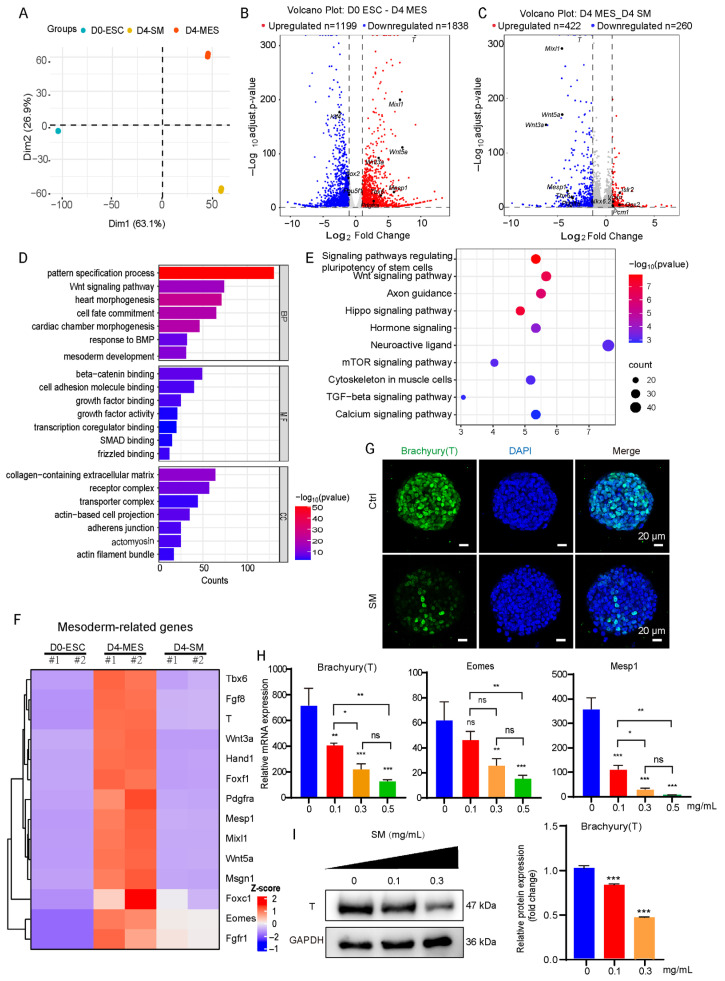
SM inhibits the development of the mesoderm. (**A**) PCA of transcriptomes from D0 ESC, day 4 mesoderm (D4-MES), and SM-treated cells (D4-SM). (**B**) Volcano plots of differentially expressed genes in D0-ESC vs. D4-MES. (**C**) Volcano plots of differentially expressed genes in D4-MES vs. D4-SM. (**D**) GO analysis of genes upregulated in the D4-MES vs. D0-ESC group via RNA sequencing (|log2FC| ≥ 1, *p* < 0.05). BP (Biological Process), CC (Cellular Component), MF (Molecular Function). (**E**) KEGG enrichment of genes upregulated in D4-MES vs. D0-ESC. (**F**) Heatmap of mesoderm-associated genes of different groups. (**G**) Immunofluorescence of Brachyury (T) on day 4. Scale bar: 20 μm. (**H**) Relative mRNA expression of *Brachyury*, *Eomes* and *Mesp1* (*n* = 3). (**I**) Protein expression levels of Brachyury(T) detected by Western blot analysis and corresponding quantification. Data are mean ± S.D (*n* = 3). ns, not significant, * *p* < 0.05, ** *p* < 0.01, *** *p* < 0.001.

During embryonic development, cardiomyocyte precursors originate from the mesodermal layer and differentiate to the myocardium [35].To evaluate how SM affects mesodermal differentiation, we performed RNA sequencing on mESCs after four days of mesoderm induction (D4-MES) and on cells subjected to the same induction protocol with SM treatment (D4-SM). Principal component analysis (PCA) separated the three groups clearly, the D4-SM samples occupied distinct position between pluripotent D0-ESCs and the properly differentiated D4-MES cells, indicating that SM induces a transcriptional state that diverges from canonical mesoderm (Figure 3A). Comparison of D0-ESCs and D4-MES by volcano plot identified 1199 significantly upregulated and 1838 significantly downregulated genes (Figure 3B). Gene Ontology (GO) analysis showed that “mesoderm development”, “response to BMP”, and “Wnt signaling pathway” were among the most significantly upregulated processes and pathways (Figure 3D,E). The Kyoto Encyclopedia of Genes and Genomes (KEGG) pathway analysis revealed the coordination of several signaling pathways, including Wnt, TGF-β, and Hippo, which are critical for exiting pluripotency and forming mesoderm (Figure 3E). This is consistent with the previous results of RT-qPCR (Figure S1F).

The volcano plot comparing D4-MES and D4-SM revealed 422 significantly up-regulated genes and 260 significantly downregulated genes in the SM-treated population (Figure 3C). These down-regulated genes are significantly associated with mesodermal development. Previous studies have confirmed that SM effectively promotes the differentiation of induced pluripotent stem cells into neurons [36]. We found that significantly upregulated genes such as *Islr2*, *Vcan*, *Gsx2*, and *Pcm1* are also associated with neuronal cell development. A heatmap focused on mesoderm-related genes demonstrated significant suppression of key lineage specifiers in SM-treated cells (Figure 3F). Notably, transcription factors such as *Brachyury (T)*, *Mesp1*, and *Eomes*, essential for mesoderm initiation and patterning, were substantially downregulated. Immunofluorescence and Western blotting indicated a dramatic, dose-dependent decrease in T protein expression (Figure 3G,I), while RT-qPCR confirmed the marked downregulation of *T*, *Mesp1*, and *Eomes* mRNA (Figure 3H). This comprehensive genomic and biological evidence underscores that SM disrupts the core regulatory network necessary for mesodermal fate acquisition.

### 3.4. SM Promotes Cardiac Progenitor Cell Transition

**Figure 4:**
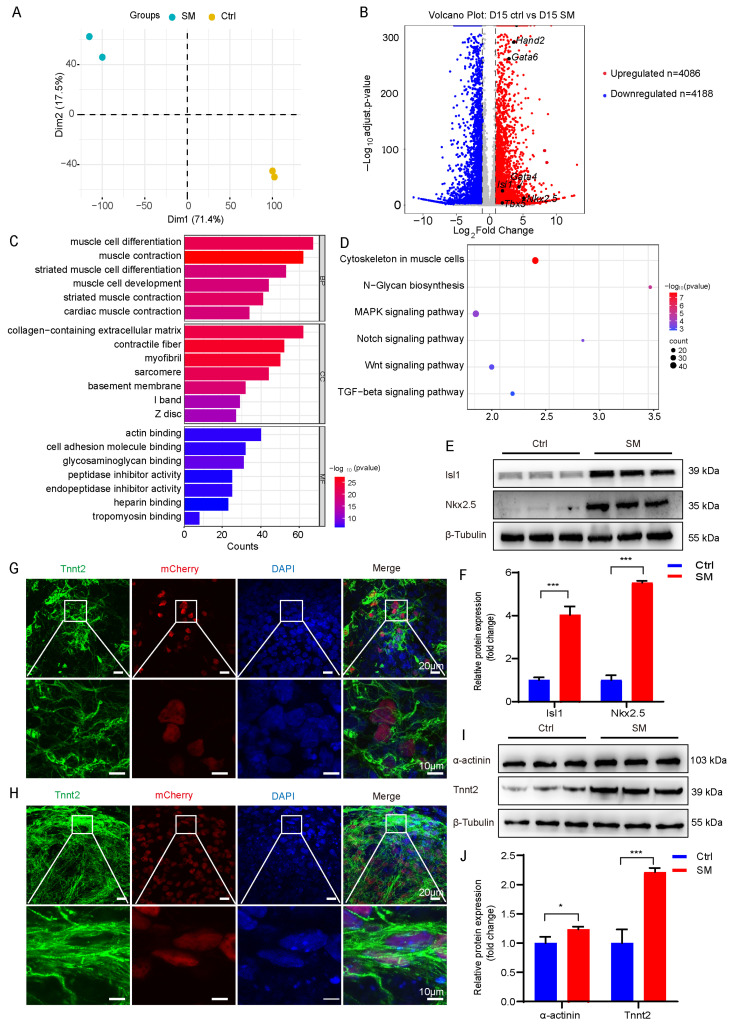
SM promotes cardiac progenitor cell fate. (**A**) PCA of day 15 transcriptomes. (**B**) Volcano plot of DEGs from control and SM treated group. (**C**) GO analysis of genes upregulated in control and SM treated group (|log2FC| ≥ 1, *p* < 0.05). BP (Biological Process), CC (Cellular Component), MF (Molecular Function). (**D**) KEGG enrichment of upregulated genes after SM treatment. (**E**) Protein expression levels of Isl1 and Nkx2.5 detected by Western blot analysis. (**F**) Corresponding quantification of Isl1 and Nkx2.5 (*n* = 3). (**G**) *Tnnt2* immunofluorescence in control group. Scale bars: 20 μm (main), 10 μm (insets). (**H**) *Tnnt2* immunofluorescence in SM treated group. Scale bars: 20 μm (main), 10 μm (insets). (**I**) Protein expression levels of *Tnnt2* and α-actinin. (**J**) Corresponding quantification of *Tnnt2* and α-actinin. Data are mean ± S.D (*n* = 3). * *p* < 0.05, *** *p* < 0.001.

After observing the suppression of early mesodermal markers, we examined the fate of SM-treated cells at a later developmental stage. Principal component analysis of the transcriptome revealed a distinct separation between SM-treated and control cells, signifying a unique cellular identity (Figure 4A). The volcano plot comparing D15-Ctrl and D15-SM highlighted significantly regulated genes (Figure 4B). GO and KEGG pathway analyses of SM-upregulated genes indicated significant enrichment in terms related to muscle development, cardiac function, and extracellular matrix organization (Figure 4C,D).

*Nkx2.5* and *Isl1* are key cardiac-specific transcription factors that begin expression during the early stages of heart formation. [37,38] The upregulation of crucial cardiac progenitor markers, such as the transcription factors *Isl1* and *Nkx2.5*, suggests that SM may influence the cardiac progenitor cell stage. To investigate this, we conducted Western blot analysis on D10 cell samples treated with and without SM, focusing on the protein expression levels of Isl1 and Nkx2.5. Our findings revealed that the group treated with SM exhibited a significant increase in the protein expression of Isl1 and Nkx2.5 compared to the control group (Figure 4E,F). This is consistent with the observed enhancement of cardiomyocyte differentiation by adding SM during the early differentiation process from D6 to D10. Furthermore, compared with the control group (Figure 4G), the robust activation of the *Tnnt2*-H2B-mCherry reporter, in the context of the co-expression of *Tnnt2* cardiac markers, confirms that SM promotes the expression within a bona fide cardiac differentiation program (Figure 4G–J). We conclude that CPC is the key stage in which SM promotes the differentiation of mESCs into cardiomyocytes.

### 3.5. SM Orchestrates Cell Fate Transition by Suppressing the Wnt/β-Catenin Signaling Pathway

**Figure 5:**
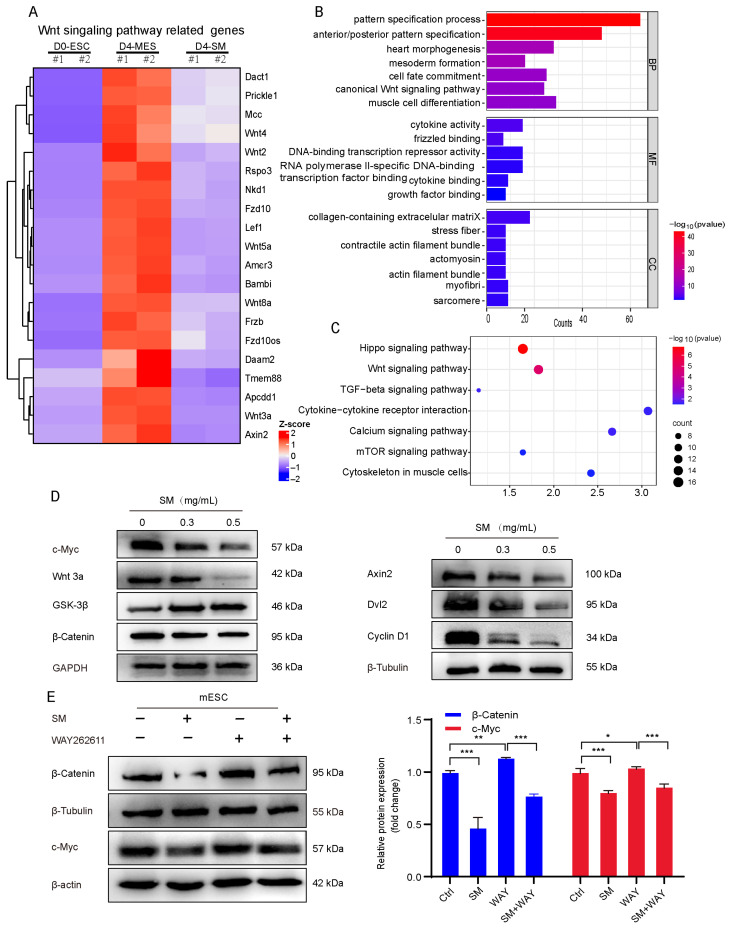
SM orchestrates cell fate transition by suppressing the Wnt/β-Catenin signaling pathway. (**A**) Heatmap of Wnt-related gene expression in different groups. (**B**) GO analysis of downregulated genes by SM on day 4. (**C**) KEGG enrichment of downregulated genes by SM on day 4. (**D**) Protein expression levels of Axin2, Dvl2, Cyclin D1, c-Myc, Wnt 3a, GSK-3β and β-Catenin. (**E**) Protein expression levels of β-Catenin and c-Myc in mESCs after treatment with SM and/or WAY262611 (WAY) detected by Western blot analysis and corresponding quantification. Data are mean ± S.D (*n* = 3). * *p* < 0.05, ** *p* < 0.01, *** *p* < 0.001.

Our transcriptomic data from Figure 3 and Figure 4 identify the Wnt signaling pathway as a primary target of SM. Wnt-related genes heatmap shows extensive downregulation in SM-treated cells compared to mesodermal controls (Figure 5A). GO and KEGG analyses corroborate the significant inhibition of the “canonical Wnt signaling” and “Wnt signaling pathway” (Figure 5B,C). Western blot analysis revealed that SM treatment decreased the protein levels of Dishevelled 2 (Dvl2), an upstream positive regulator of β-catenin, and caused a dose-dependent reduction in total β-catenin levels (Figure 5D). Additionally, the expression of Wnt/β-catenin target genes, such as Axin2, c-Myc, and Cyclin D1, were significantly suppressed (Figure 5D). To further elucidate the underlying mechanism, we investigated whether activating the wnt pathway could counteract SM’s effects. Co-treatment with SM and the Wnt activator WAY262611 restored the protein levels of active β-catenin and c-Myc (Figure 5E). This experiment provides conclusive evidence that the inhibition of the canonical Wnt/β-catenin pathway is the underlying mechanism for SM’s effects on cell cycle exit and differentiation.

### 3.6. Tanshinone IIA Is Identified as the Most Potent Pro-Cardiogenic Compound Derived from SM

**Figure 6:**
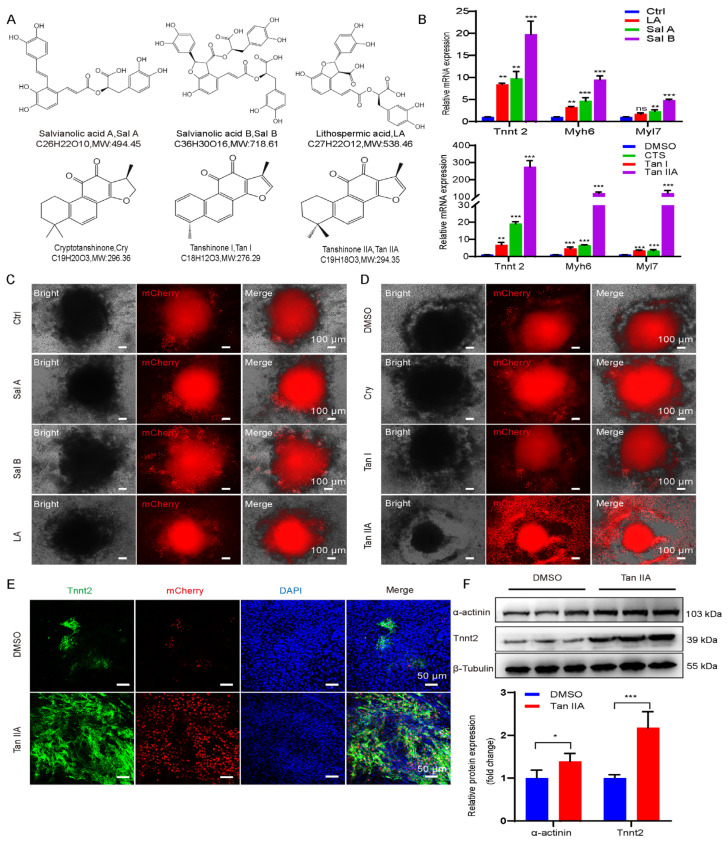
Tan IIA is identified as the most potent pro-cardiogenic compound derived from SM. (**A**) Chemical structures of six major SM compounds used in this study. (**B**) Relative mRNA expression of *Tnnt2*, *Myh6*, and *Myl7*. The concentrations of *Salvia miltiorrhiza* Bunge compounds used during differentiation were Sal A, 20 μg/mL; Sal B, 10 μg/mL; LA, 20 μg/mL; Tan I, 0.5 μg/mL; Tan IIA, 20 μg/mL; Cry, 20 μg/mL. (**C**) Representative images of Sal A, Sal B, and LA screening using the *Tnnt2* reporter line. Scale bar: 100 μm. (**D**) Representative images of Cry, Tan I and Tan IIA screening using the *Tnnt2* reporter line. Scale bar: 100 μm. (**E**) Representative immunofluorescence images of *Tnnt2* staining treated with DMSO and Tan IIA. mCherry (red)/DAPI (blue), Scale bar: 50 μm. (**F**) Protein expression levels of *Tnnt2* and α-actinin detected by Western blot analysis and corresponding quantification. Data are mean ± S.D (*n* = 3). ns, not significant, * *p* < 0.05, ** *p* < 0.01, *** *p* < 0.001 vs. 0.1% DMSO control.

To identify the active component responsible for the observed cardiac induction, we screened a panel of major compounds derived from SM using our *Tnnt2*-H2B-mCherry reporter line. Among both lipophilic tanshinones and hydrophilic salvianolic acids, Tanshinone IIA (Tan IIA) emerged as the most potent inducer of the cardiac reporter (Figure 6A–D). The treatment with Tan IIA consistently led to the synchronous contraction of the entire EB (Videos S1 andS2). Treatment with Tan IIA resulted in the strongest activation of *Tnnt2* expression (Figure 6B,D) and also significantly upregulated other key cardiac markers, including *Myh6* (*α-MHC*) and *Myl7* (*MLC2a*), at the mRNA level (Figure 6B). This effect was confirmed at the protein level, with Tan IIA-treated cells showing a substantial increase in *Tnnt2* and α-actinin protein expression (Figure 6E,F). We thus identify Tan IIA as the primary bioactive compound in SM that promotes cardiomyocyte differentiation.

### 3.7. Tanshinone IIA Recapitulates the SM Function by Suppressing the Wnt/β-Catenin Pathway to Promote Cardiomyocyte Differentiation

**Figure 7:**
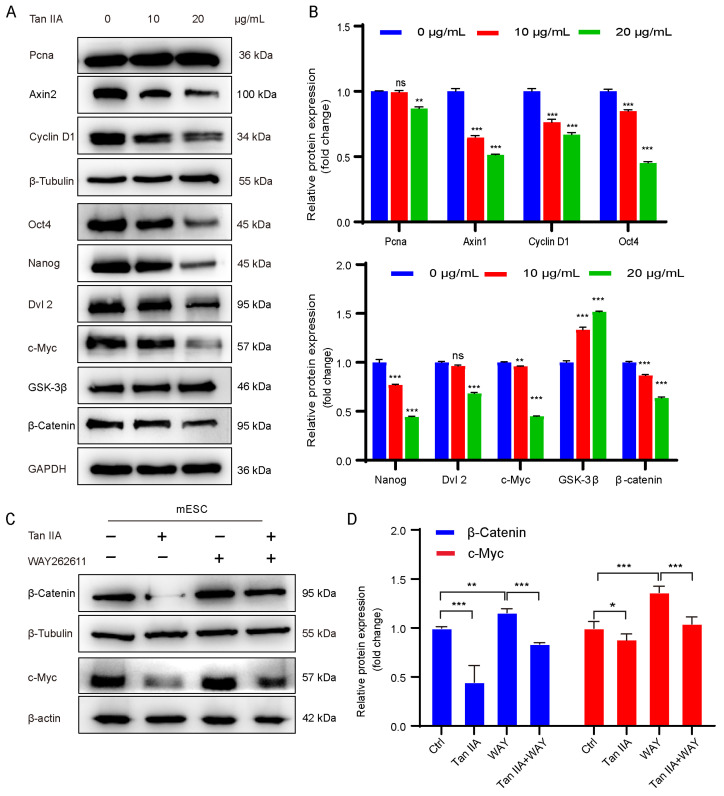
Tan IIA recapitulates the SM function by suppressing the Wnt/β-Catenin pathway to promote cardiomyocyte differentiation. (**A**) Protein expression levels of pluripotency, proliferation, and Wnt pathway genes after Tan IIA treatment detected by Western blot analysis. (**B**) Corresponding quantification of protein expression levels (*n* = 3). (**C**) Protein expression levels of β-Catenin and c-Myc in mESCs after treatment with Tan IIA and/or WAY262611 (WAY). (**D**) Corresponding quantification of protein expression levels β-catenin and c-Myc in mESCs after treatment with Tan IIA and/or WAY262611. Data are mean ± S.D (*n* = 3). ns, not significant, * *p* < 0.05, ** *p* < 0.01, *** *p* < 0.001.

We next tested whether the active compound Tan IIA acts by the same mechanism as SM treatment. In mESCs, Tan IIA induced a dose-dependent reduction in core pluripotency factors Oct4 and Nanog and proliferation marker Pcna. At the same time, Tan IIA decreased protein levels of key Wnt/β-catenin pathway components, including Dvl2, β-catenin, and the downstream targets c-Myc and Cyclin D1 (Figure 7A,B). Importantly, the Wnt pathway activator WAY262611 restored β-catenin and c-Myc expression, counteracting the suppressive effects of Tan IIA (Figure 7C,D). These findings indicate that Tan IIA is the primary active component driving the observed phenotype and that it acts by selectively inhibiting canonical Wnt/β-catenin signaling.

## 4. Discussion

The regeneration of functional cardiomyocytes from PSCs presents significant potential for the treatment of myocardial infarction and heart failure. This study systematically elucidates the temporal sequence and molecular mechanisms by which SM and its active component, Tan IIA, regulate the differentiation of mESCs into cardiomyocytes. We demonstrated that SM and Tan IIA regulate cell fate by precisely coordinating the exit from pluripotent states, inhibiting early mesodermal development, and promoting the differentiation of CPCs through the suppression of the classical Wnt/β-catenin signaling pathway.

The transition from a pluripotent state to a differentiated lineage necessitates the regulation of the core transcriptional network. Our data demonstrate that treatments with SM and Tan IIA effectively inhibit pluripotency markers, including *Oct4*, *Sox2*, and *Nanog*. This aligns with previous research on the modulation of pluripotent networks by natural compounds such as resveratrol and icariin [39,40,41]. Furthermore, it corroborates earlier reports regarding the inhibitory effects of SM and Tan IIA on the proliferation of human placental mesenchymal stem cells [23,42]. By inducing a stall at this stage, SM may effectively lower the differentiation threshold. In contrast to nonspecific cytotoxic agents that trigger widespread apoptosis, SM selectively reduces *Pcna* and *Cyclin A* expression. This cell cycle inhibition creates a conducive cellular environment for subsequent differentiation, unlike the potent cytotoxic effects of conventional differentiation inducers like 5-azacytiabine, highlighting the benefits of incorporating traditional Chinese medicine compounds in stem cell modulation [43,44,45].

The activation of the Wnt signaling pathway leads to the accumulation of β-catenin in the cytoplasm and facilitates its translocation to the nucleus. Within the nucleus, β-catenin interacts with TCF/LEF family proteins to form a transcriptional complex, which subsequently activates downstream genes, including *c-Myc* and *cyclin D1* [46]. In contrast, in the absence of Wnt signaling, the CK1α/APC/Axin/GSK-3β complex phosphorylate β-catenin. This phosphorylated β-catenin is recognized by β-TrCP, leading to its ubiquitination and eventual degradation by the proteasome [16,17,47]. Our analyses, including transcriptomics, immunofluorescence, Western blot, and RT-qPCR, consistently identified the Wnt/β-catenin pathway as the primary target of SM. Treatment with SM reliably down-regulates Dvl2, β-catenin, and their downstream targets, such as Axin2, c-Myc, and cyclin D1. These findings suggest that the inhibition of Wnt/β-catenin signaling may represent the central mechanism through which SM its effects. Recovery experiments utilizing the Wnt activator WAY262611 further corroborated the causal relationship between Wnt inhibition and the effects of SM. Notably, transcriptome analyses revealed changes in the expression of *Wnt11* and *Wnt5a*, both of which are integral components of the atypical Wnt signaling pathway and play essential roles in cardiac development and function [48,49,50]. This also emphasizes the necessity for precise regulation of the Wnt/β-catenin signaling pathway during cardiac differentiation [14,47,51,52,53].

A key finding of this study is that SM exerts completely opposite effects at different stages of differentiation. This time-dependent effect aligns closely with the biphasic mode of action of the Wnt/β-catenin pathway during myocardial differentiation [14,52]. Research conducted over the past few decades has elucidated the critical mechanisms through which the Wnt/β-catenin signaling pathway regulates cell proliferation and fate determination throughout embryonic development, adult homeostasis, and tissue regeneration [15,17,51]. The classical Wnt/β-catenin signaling pathway is essential for blastoderm formation and mesoderm induction; however, it must subsequently be inhibited to facilitate the differentiation of the mesoderm into cardiac progenitor cells. SM mimics the dynamic changes in endogenous Wnt signaling that occur during embryonic heart development by sequentially inhibiting this pathway. This mechanism resonates with established small molecule differentiation protocols, which also utilizes Wnt inhibitors like IWP-2, IWP-4 and XAV939 during the second stage of differentiation [14,52,54]. This temporal specificity underscores the significance of targeted interventions at specific developmental phases in stem cell manipulation and partially elucidates the mechanism behind *Salvia miltiorrhiza* Bunge’s potential in enhancing cardiac regeneration clinically, likely through its interaction with resident progenitor cells.

Through systematic screening of the primary lipophilic and hydrophilic components in SM, we identified Tan IIA as the principal bioactive component that promotes myocardial formation. Its efficacy surpasses that of hydrophilic (water-soluble) phenolic compounds and certain non-polar (lipophilic) diterpenoids. This finding aligns with the pharmacological research conducted by Li et al., which focused on identifying the effective components of SM that facilitate the differentiation of human placental mesenchymal stem cells into cardiomyocytes [24,42]. Furthermore, Tan IIA replicated the biological effects of SM by inhibiting pluripotency markers, suppressing cell proliferation, and promoting cardiac gene expression. It specifically blocks the Wnt/β-catenin pathway, consistent with pharmacological studies that emphasize the role of Tan IIA in regulating the physiological functions of blood vessels and the heart through various pathways [21,55,56].

Despite the encouraging findings, several limitations persist. While mESCs serve as valuable research models, notable differences exist among species. The translational potential of Tan IIA must be validated in human induced pluripotent stem cells (hiPSCs) to confirm its clinical relevance in regenerative medicine Second, although we have identified the Wnt pathway as the primary target, the specific molecular binding sites of Tan IIA remain unclear. It is uncertain whether Tan IIA directly targets the Frizzled receptor, disrupts the stability of the degradation complex, or interacts directly with β-catenin. Additionally, transcriptomic analyses indicate alterations in the TGF-β, BMP, and Hippo signaling pathways. Given the extensive cross-regulation between these pathways and Wnt signaling during cardiac development, future studies should investigate whether Tan IIA produces a synergistic effect within these signaling networks. Finally, although we have achieved high differentiation efficiency, the electrophysiological maturity of cardiomyocytes derived from stem cells still requires further investigation through patch clamp or multi-electrode array (MEA) systems. Future research should also assess the in vivo implantation potential of these cells in myocardial infarction models to evaluate their functional recovery and tissue integration capabilities.

## 5. Conclusions

Our study presents a detailed framework for understanding how SM and Tan IIA regulate cardiac differentiation by influencing the Wnt/β-catenin pathway over time. These findings bolster the scientific validity of traditional Chinese medicine and introduce an innovative, natural product-based approach for generating cardiomyocytes, which is valuable for both regenerative medicine and drug screening applications.

## Figures and Tables

**Table 1:** Primers for sgRNA and PCR suitable for the *Tnnt2* H2B-mCherry reporter cell line.

Name	Primer Sequence (5′ to 3′)	Product Size (bp)
*Tnnt2* sgRNA	F ^1^-gCTCGGCGTCAGACATGCTCT	20
	R ^1^-AGAGCATGTCTGACGCCGAGc	
*Tnnt2* F1 + R1	F-CAGTCCCTGTTCAGAGGTAAGACA	5649
	R-TAAGATACATTGATGAGTTTGG	
*Tnnt2* F2 + R2	F-GCTGTGCTCGACGTTGTCAC	4863
	R-GTGACAGGACATCAAGACTCACTG	
*Tnnt2* F3 + R3	F-CTACCGATCTTGAGTTTGTCACA	2192
	R-CAGGGTGGACCTGCTTCAGAACCT	
*Tnnt2* F4 + R4	F-CCGCTTCCTCGTGCTTTACG	740
	R-CGGCAGCTCCAAGGAAAA	

^1^ F, forward; R, reverse.

**Table 2:** RT-qPCR primers used in this study.

Name	Primer Sequence (5′ to 3′)	Product Size (bp)
*β-actin*	F-TAGGCACCAGGGTGTGATGG	282
	R-ATGGCTGGGGTGTTGAAGG	
*Oct4*	F-GGCTAGAGAAGGATGTGGTTCGAG	118
	R-CCTGGGAAAGGTGTCCCTGTAG	
*Nanog*	F-TGAGCTATAAGCAGGTTAAGAC	136
	R-CAATGGATGCTGGGATACTC	
*Sox2*	F-CGGCACAGATGCAACCGAT	86
	R-CCGTTCATGTAGGTCTGCG	
*P* *cna*	F-TTTGAGGCACGCCTGATCC	135
	R-GGAGACGTGAGACGAGTCCAT	
*Cyclin A*	F-TGGCTGTGAACTACATTGA	136
	R-ACAAACTCTGCTACTTCTGG	
*Brachyury*	F-CTCGGATTCACATCGTGAGAG	148
	R-AAGGCTTTAGCAAATGGGTTGTA	
*Mesp1*	F-TGTACGCAGAAACAGCATCC	135
	R-TTGTCCCCTCCACTCTTCAG	
*Eomes*	F-TGTGACGGCCTACCAAAACA	112
	R-ACCTCCAGGGACAATCTGATG	
*Isl1*	F-AAGGACAAGAAACGCAGCAT	85
	R-TTCCTGTCATCCCCTGGATA	
*Gata4*	F-CTCTATCACAAGATGAACGGCATCAAC	100
	R-TCTGGCAGTTGGCACAGGAGAG	
*Tnnt2*	F-GTAGAGGACACCAAACCCAAG	139
	R-GAGTCTGTAGCTCATTCAGGTC	
*Myh6*	F-GATGCCCAGATGGCTGACTT	275
	R-GGTCAGCATGGCCATGTCCT	
*Myl7*	F-CCCATCAACTTCACCGTCTTCCT	167
	R-AGAGAACTTGTCTGCCTGGGTCA	

## Data Availability

The original code has been deposited at CNCB under accession number PRJCA058435 and at the Gene Expression Omnibus (GEO) repository under accession number PRJNA1244115.

## Supplementary Materials

The following supporting information can be downloaded at: https://www.mdpi.com/article/10.3390/cells15090786/s1, Figure S1. Generation and validation of the *Tnnt2*-H2B-mCherry knock-in reporter mESC line. Figure S2. SM suppresses mESC proliferation. Video S1: Tan IIA-treated EB of reporter cell line showing the contracting area (Bright); Video S2: Tan IIA-treated EB of reporter cell line showing the contracting area(mCherry).

## Abbreviations

AP	Alkaline Phosphatase
CPC	Cardiac Progenitor Cell
CM	Cardiomyocyte
EB	Embryoid Body
EdU	Ethynyl-labeled Deoxyuridine
GO	Gene Ontology
GSK-3β	Glycogen Synthase Kinase-3 Beta
IF	Immunofluorescence
iPSC	Induced Pluripotent Stem Cell
KEGG	Kyoto Encyclopedia of Genes and Genomes
Isl1	LIM Homeobox 1
mESCs	Mouse Embryonic Stem Cells
Nkx2.5	NK2 Homeobox 5
Oct4	Octamer Binding Protein-4
Tan IIA	Tanshinone IIA
*Tnnt2*	Troponin T Type 2
WB	Western Blotting
WT	Wild type
