# Supplementary material for: Salvia miltiorrhiza Bunge Regulates the Differentiation of mESCs into Cardiomyocytes via the Wnt/β-Catenin Signaling Pathway
Source: Cells. 2026 Apr 26;15(9):786. doi: 10.3390/cells15090786 (PMC13163000; doi:10.3390/cells15090786)
Supplement: Supplementary file 1 [file cells-15-00786-s001.zip › Figure S1 and S2.pdf]

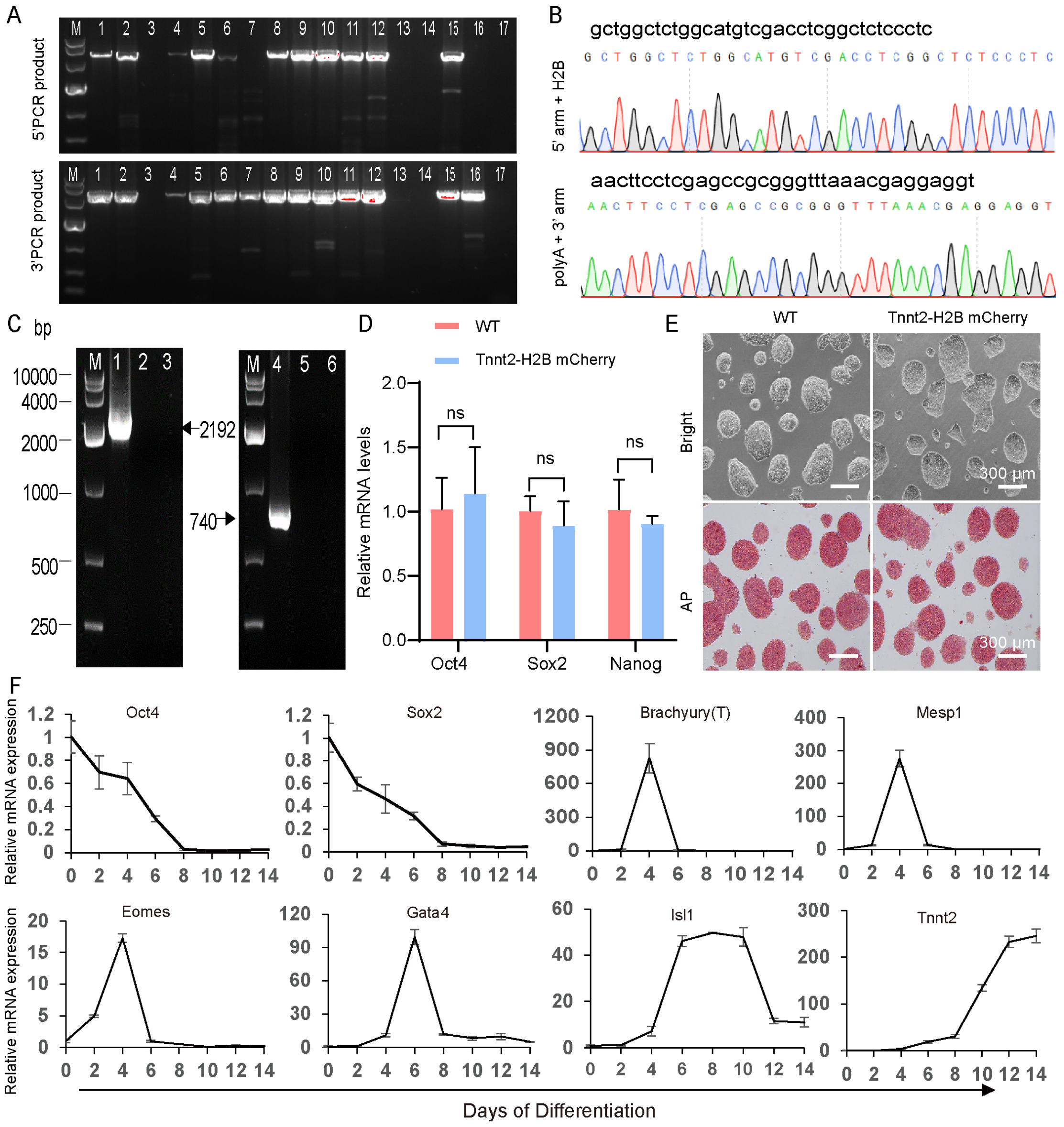

Figure.S1. Generation and validation of the Tnnt2-H2B-mCherry knock-in reporter mESC line. (A) PCR genotyping of reporter cell line. (B) Sanger sequencing of homologous arms. (C) PCR for Positive clone. (D) Relative mRNA expression of pluripotency genes in WT and reporter lines. (E) Representative images of phase-contrast and AP staining. Scale bar: 300 μm. (F) Relative mRNA expression levels during differentiation. Data are mean ± S.D. ns, not significant.

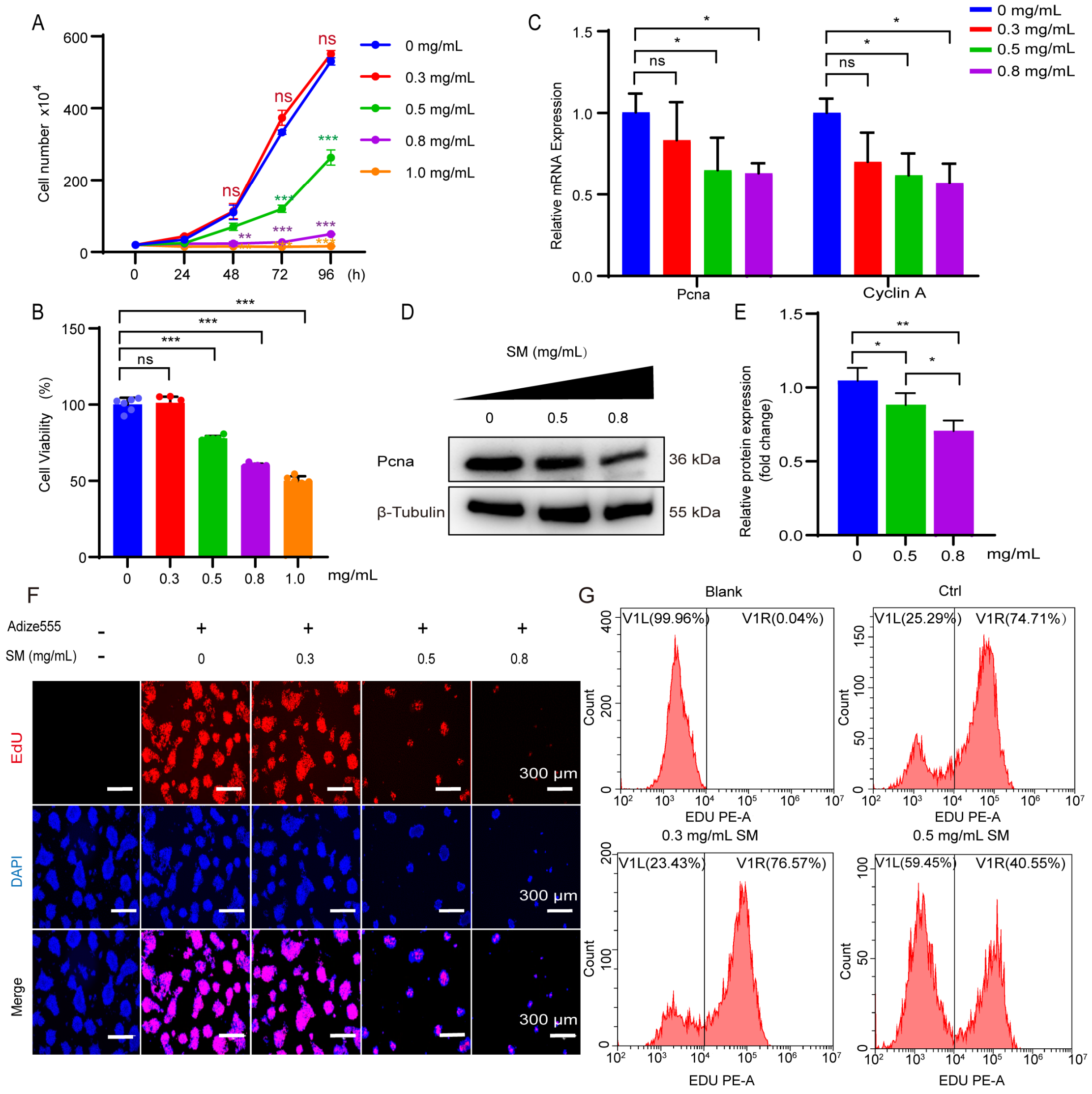

Figure.S2. SM suppresses mESC proliferation. (A) Cell counts after SM treatment (*n*=3). (B) MTT assay for cell viability (*n*=6). (C) Relative mRNA expression of *Pcna* and *Cyclin A* (*n*=3). (D) Protein expression levels of Pcna detected by western blot analysis. (E) Corresponding quantification of Pcna protein(*n*=3). (F) Representative images of EdU incorporation assay of different groups. Scale bar: 300 μm. (G) Flow cytometry analysis of EdU⁺ cells.
